# A novel approach to non-segmented flow analysis: Part 4. Aluminium in river waters

**DOI:** 10.1155/S1463924691000287

**Published:** 1991

**Authors:** D. J. Malcolme-Lawes, K. H. Wong

**Affiliations:** Centre for Research in Analytical Chemistry and Instrumentation King's College London Strand London WC2R 2LS UK

## Abstract

A rapid and precise method is developed for the determination of aluminium in water. The results demonstrate that the calibration range of the assay can be extended by a simple manipulation of the control program of the flow analyser. An RSD of 1.8% is achieved for injection of standards and the theoretical limit of detection is estimated at 0.33 ppm alum (equivalent to 18 ppb Al^3+^). The method is applied to monitoring of environmental samples.


Journal of Automatic Chemistry, Vol. 13, No. 4 (July–August 1991), pp. 147–151

### A novel approach to non-segmented flow analysis: Part 4. Aluminium in river waters

**D. J. Malcolme-Lawes and K. H. Wong**

*Centre for Research in Analytical Chemistry and Instrumentation, King's College London, Strand, London WC2R 2LS*

*A rapid and precise method is developed for the determination of aluminium in water. The results demonstrate that the calibration range of the assay can be extended by a simple manipulation of the control program of the flow analyser. An RSD of 1·8% is achieved for injection of standards and the theoretical limit of detection is estimated at 0·33 ppm alum (equivalent to 18 ppb $Al^{3+}$). The method is applied to monitoring of environmental samples.*

### Introduction

Aluminium is the third most abundant element on the Earth's surface. It was brought to the public's attention recently, when, in 1988, a significant quantity of aluminium sulphate (alum) was mistakenly discharged into the water supply system to the village of Camelford in Cornwall. Local residents soon complained of adverse discomfort and hair discolouration. Alum, at more appropriate levels, is added to natural waters to remove impurities from peaty soils and to reduce treatment necessary by chlorination. Although it has been extensively reported in the media that aluminium is the probable cause of Alzheimer's disease, a recent report by the Aluminium Association in the USA [1] has concluded that the cause of Alzheimer's disease is not known and ordinary environmental exposure to aluminium is safe. It should be noted that only 4% of the total exposure of aluminium to humans comes from natural waters and over 90% of it comes from food. Aluminium is leached from the soil by acidic precipitation, the mechanism of ionic transport is studied by Driscoll and Schecher [2].

Instrumental methods which are most widely used for the determination of aluminium include atomic absorption spectroscopy, inductively coupled plasma emission spectroscopy and neutron activation analysis. Photometric techniques for its determination are widely available. However, they rely on the reaction of a complexing agent with $Al^{3+}$, the latter is susceptible to hydrolysis in aqueous solutions. Below pH 5, the $Al^{3+}$ ion predominates, this slowly hydrolyses as the pH increases. At neutral pH, it was originally thought that soluble aquo-species of aluminium did not exist and they could only be stabilized by complexation or the formation of insoluble hydroxides [3]. Bertsch [4] conducted a detailed study of the hydrolytic products of aluminium and concluded that around neutral pH there exists a number of species, particularly polynuclear aluminium complexes, which are metastable in aqueous solutions. The actual structure and solution chemistry of these species are too complicated to be included within the scope of this study. Therefore, the determination of aquo-, as opposed to hydroxyl-, species of aluminium should be performed at or below pH 5, whereby their concentrations increase exponentially with decreasing pH. Another peril to aluminium determinations is the existence of complexing ligands which compete with aquo-ligands for the central Al atom. Species which form strong complexes with aluminium include hydroxide and fluoride.

The number of organic reagents given in the literature that are suitable for the determination of aluminium photometrically is staggering, Burger [5] listed no less than 21 such chromogenic reagents in his book. Of those listed, only a handful exhibit high selectivity for only one or two metals, including aluminium, examples are aluminon, chrome azurol S (CAS), eriochrome cyanin R (ECR) and stilbazo. Up to the early 1970s, the reagent that was most widely used for the determination of aluminium in Britain was 'aluminon'. It was favoured for the stability of its coloured product and its wide pH tolerance range, but it suffered from such disadvantages as non-linear calibrations and heating for full colour development. Aluminon, together with other commonly used organic reagents, were comprehensively reviewed by Dougan and Wilson [6]. In one of the papers being reviewed, Pakalns compared the use of CAS, ECR and aluminon and concluded that CAS was the best on the grounds of simplicity, selectivity and reliability. In spite of this, Dougan and Wilson themselves discarded the use of CAS due to its high blank values and opted for pyrocatechol violet (PCV). Zhu *et al.* [7] highlighted the importance of pH as most of the organic reagents possess an extremely narrow optimum pH range. Marczenko and Jarosz [8] compared the use of three organic reagents: ECR, CAS and PCV in conjunction with various cationic surfactants and demonstrated that the addition of a third component, i.e. the surfactant, could enhance the sensitivity of aluminium determinations. They also calculated the stoichiometry of the ternary complexes. Liu [9] studied the effect of adding ethanol to a ternary system and observed a three-fold increase in the absorbance value in the presence of between 25 and 40% ethanol compared with no ethanol at all. Sampson and Fleck [10] again compared the use of some organic reagents with cationic surfactants and they used a CAS/Cetylpyridinium chloride (CPC) system for the determination of aluminium in dialysis fluids and water. Many of these methods have been adapted to flow injection analysis (FIA) systems and applied to a variety of sample matrices. The Brazilian pioneers in FIA, Reis *et al.*, determined aluminium in soil and plant digest [11]. Wyganowski *et al.* used a combined reagent of bromopyrogallol red (BPR), n-tetradecyltrimethylammonium bromide (TDTA) and ethanol in a hexamine buffer using a dual-line manifold for analysing river water [12]. Royset compared the performance of some organic

0142–0453/91 $3.00 © 1991 Taylor & Francis Ltd.

reagents in the presence and absence of a surfactant in FIA [13] and adapted a system based on PCV [14]. Bouzid and Macdonald [15] used the CAS/CPC/ethanol combination and achieved phenomenal sensitivity. A comparison chart is also provided for various ternary complexes of aluminium. Henshaw *et al.* [16] used a system, not dissimilar to the one employed by Royset, for the determination of 'monomeric aluminium species' in natural waters.

### Experimental

The aim of our experiments was to devise a method for the routine monitoring of water for aluminium in the form of alum. Water authorities discharge around 20 ppm of alum into water supplies at source to clarify the water. Hence, a calibration of up to 20 ppm of alum is required, corresponding to 1·13 ppm of Al.

The flow analyser has remained virtually unchanged from that previously described [17]. The combined reagent comprising CAS/CPC/ethanol was chosen as it has proven to be sensitive, selective, versatile (in terms of pH tolerance) and possess a good linear range. The stock reagent solutions were prepared by dissolving 1·08 g of CAS and 0·64 g of CPC in separate litre flasks. The stock masking agent comprised of 100 g of hydroxylammonium chloride (HOAC) with 1·0 g of 1,10-phenanthroline monohydrate (PAL) per litre. The stock buffer solution contained 420·57 g of hexamine with 50 ml of 37% hydrochloric acid per litre. Both the masking agent and the buffer solution were prepared only when required. The stock alum standard solution was prepared by dissolving 1·00 g of aluminium potassium sulphate dodecahydrate in 1 litre. Working solutions of the standard were prepared upon dilution of the stock standard giving solutions containing 5, 10, 15 and 20 ppm of alum standards. All the solutions were prepared in de-ionized water which was filtered through a 0·45 micron filter and de-gassed using nitrogen. A combined reagent system was prepared by mixing the following stock solutions: 10 ml CAS + 20 ml CPC + 3 ml masking agent + 3 ml buffer and 30 ml ethanol. The constituents were added in the order stated and the mixture was allowed to cool before topping up to 100 ml with water.

This reagent system had a pH of 5·0 ± 0·1 and was stable for at least 24 hours. A pH of 5·0 was selected to ensure that the species analysed would be predominantly monomeric $Al^{3+}$, it also fell conveniently into the optimum pH range for the CAS/CPC combination [8]. Such high concentrations for the dye and surfactant were required as we elected to operate in the region of optimum stoichiometry of the Al-CAS-CPC ternary complex. The minimum two-fold excess of CAS and four-fold excess of CPC over $Al^{3+}$ was far exceeded. Inevitable consequences were relatively high blank values caused by a substantial reagent peak. A simple method was devised to tackle this particular problem.

In a previous paper [17] the authors reported on the determination of a wide range of analyte concentration by incorporating an extra valve into the manifold to perform in-line dilution of the sample reagent mixture, which had

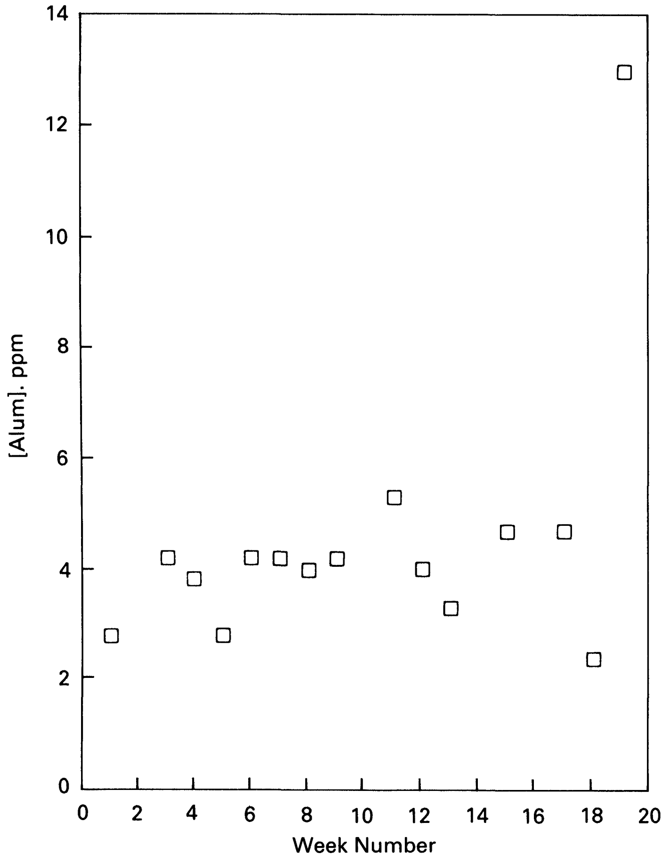

*Figure 1. Variation of alum concentration with time.*

the effect of substantially extending the calibration range available. Although this method was shown to be successful, the authors have now developed a technique which requires no modification to the flow pattern, and so may be used to record usable data without prior knowledge of the analyte concentration range within the sample. A simple modification to the software overcomes the problem of high blank values and extends the usable calibration range.

Tyson [18] studied the phenomenon of reagent dispersion in a single-line manifold and explained the occurrence of double peaks, generated when the reagent and the sample components became inter-dispersed. In the authors' experiments, the sample-reagent mixture peak at low sample concentrations appeared as a small peak along the leading edge of the flow profile, and is followed by a larger peak caused by absorbance of the higher concentration reagent mixture. At higher sample concentrations the situation was reversed as the sample-reagent peak is much larger than the reagent peak. The time between the sample injection and the appearance of the first peak, the sample-reagent peak, was the same regardless of the concentration of the sample, as would be expected from basic FIA principles. In these experiments the product absorbance was recorded at a point before the maximum of the sample-reagent mixture peak and this value was used for the analytical measurement. This time was called the 'peak time'. This procedure ensured that the analytical measurement was derived from the sample-reagent mixture and not influenced by excess reagent absorbance.

*Table 1. Variation of calibration parameters with peak time.*

| Peak time (s) | Slope (AU/ppm) | Intercept (AU) | Corr. coeff. $N = 5$ |
|---|---|---|---|
| 2 | 0·0257 | 0·1473 | 0·987 |
| 3 | 0·0434 | 0·2483 | 0·989 |
| 4 | 0·0485 | 0·2201 | 0·995 |
| 5 | 0·0419 | 0·3141 | 0·987 |
| 6 | 0·0334 | 0·4473 | 0·983 |

At a flow rate of 5 ml min$^{-1}$, the leading edge of the sample arrived at the flow cell at less than 5 s after the injection of the sample. In order to establish the peak time, a preliminary run was conducted using de-ionized water as the sample. Various peak times were tested down to a minimum of 1 s before the maximum of the first peak. The influence of the peak time on some calibration characteristics is listed in table 1 for a series of five alum standards ranging from 0 to 20 ppm.

A peak time of 4 s was selected for subsequent experiments. Note that a smaller peak time could be selected where lower sensitivity was required. Other flow analyser parameters used for the determination of alum, in line with the terminology that has been previously described, were:

| Parameter | |
|---|---|
| Dilution factor· | 1 s |
| Wait time | 0 s |
| Fill time | 6 s |
| Wash time | 25 s |
| Reaction temperature | Ambient °C |
| Wavelength | 625 nm |
| Sample throughput | >100/h |

### Results and discussion

Under the stated conditions, the calibrations obtained for five consecutive sets of standards, covering the range 0 to 20 ppm alum, are summarized in table 2.

Having established that the calibrations were reproducible, the precision for each standard was measured and listed in table 3.

*Table 2. Calibration characteristics for a series of standards.*

| Calibration no. | Slope (AU/ppm) | Intercept (AU) | Corr. coeff. $N = 5$ |
|---|---|---|---|
| 1 | 0·0478 | 0·2203 | 0·9966 |
| 2 | 0·0485 | 0·2175 | 0·9938 |
| 3 | 0·0473 | 0·2267 | 0·9946 |
| 4 | 0·0492 | 0·2255 | 0·9935 |
| 5 | 0·0497 | 0·2105 | 0·9943 |
| Mean | 0·0485 | 0·2201 | 0·9946 |

*Table 3. Reproducibility of sample injection.*

| Sample concn. (ppm) | Peak height (mean AU) | RSD (%) |
|---|---|---|
| | | $N = 10$ |
| 0 | 0·2363 | 3·36 |
| 5 | 0·4210 | 2·95 |
| 10 | 0·7515 | 1·63 |
| 15 | 1·0205 | 0·60 |
| 20 | 1·1615 | 0·42 |
| | | Mean RSD = 1·79% |

The theoretical limit of detection (twice the standard deviation of the blank divided by the slope of the calibration) is estimated to be 0·328 ppm alum, which is equivalent to 18 ppb of $Al^{3+}$. However, none of the real samples used in this study have had alum levels close to the limit of detection.

*Interference study*

Potential interferents were investigated for their effect on 10 ppm standard solutions of alum. Commonly interfering species which are most likely to be found in potable water include: iron$^{2+}$, copper$^{2+}$, fluoride, orthophosphate and detergents. The concentration of these substances were prepared to match very closely to the concentrations normally encountered in potable water. All the substances looked at caused negative interference on the standard. The results are summarized in table 4.

Low level interference implies that the presence of these substances caused less than 10% suppression of the 10 ppm signal. Moderate implies between 10 and 20% suppression. Anything above 20% suppression is classified as extensive interference.

Fluoride interference is widely reported in the literature for the determination of Al. Due to its ionic radius, the fluoride ion has a high affinity for the central Al atom. The stability constant for the equilibrium reaction:

$$Al^{3+} + 6F^{-} <\!-\!-\!-\!> [AlF_6]^{3-} \text{ is } 6 \times 10^{19}.$$

The influence of fluoride extends from pH 4·5 to pH 6·5. Not surprisingly, the fluoride interference cannot be easily overcome. Iron interference was minimized in the conventional way. A pH of 5·0 is well within the operational range of PAL but the level of $Fe^{2+}$ causing moderate interference has exceeded the amount normally

*Table 4. Level of interference of foreign species.*

| Low | Moderate | Extensive |
|---|---|---|
| 0·1 ppm $Fe^{2+}$ | 0·1 ppm fluoride | |
| 1 ppm $Cu^{2+}$ | 1 ppm $Fe^{2+}$ | 1 ppm fluoride |
| 1 ppm orthophosphate | | |
| 1 ppm detergent* | | |
| 5 ppm detergent | | |

* Commercial Teepol.

encountered in water. For a high level of $Fe^{2+}$, the amount of masking agent should be increased to minimize the error caused.

*Analysis of water samples*

Samples of water were collected along the River Stour in Kent at 12 different sites over a period of several months for the determination of alum. Week one was given the prefix '01' and the first site was given the suffix 'A'. For example, '03F' denotes a sample gathered from site F during the third week of collection. The samples were collected over a few months to observe periodic fluctuations; the purpose of amassing samples from different sites along the river was to investigate the effect of the surroundings on the level of alum. The sites are as follows:

A: River mouth, eddy current along the bank, silty water.
B: At Sandwich, Kent, 8 feet tidal rise, muddy river bed.
C: Main drainage ditch, slow moving water, cloudy river bed.
D: Below the Minster marshes, 4 feet tidal rise, thick-mud bed.
E: Above the Ash, convergence of two rivers, muddy bed.
F: Non-tidal, light mud and weeded river bed.
G: 2·5 feet tidal rise, mud over gravel, weeded river bed.
H: One foot tidal rise, mud over gravel, weeded river bed.
I: Upstream from sewage works, shallow fast moving, weeded bed.
J: Downstream from sewage works, rapid moving water.
K: Fast moving water, no weed or silt on the river bed.

Preliminary experiments, see Chart 1, have identified a steady level of alum ranging from 2·4 to 5·3 ppm over a period of 18 weeks for samples collected at site A. However, the level rose abruptly in week 19 and this prompted further investigation for the samples collected from this week. The results are shown in Chart 2. There appears to be a correlation between the level of alum measured and the tidal rise along different stretches of the river. For example, the highest concentration was found at site B where the tidal rise was 8 feet; the alum levels rose again from C to D and from F to G where the tidal levels also increased from the preceding site. It must be stressed, however, that this correlation could not be substantiated in all cases. Concentration at site I was lower than that of site J although the latter had a tidal rise of 1 foot, perhaps because samples I and J were collected just before and just after a sewage works respectively. Despite the absence of solid correlation, an overall downward trend is observed indicating that the level of alum has been diluted gradually (or leached into the surrounding soil) along the river.

*Chart 1.*

| Samp. no. | 01A | 03A | 04A | 05A | 06A | 07A | 08A | 09A |
|---|---|---|---|---|---|---|---|---|
| Alum, ppm | 2·8 | 4·2 | 3·8 | 2·8 | 4·2 | 4·2 | 4·0 | 4·2 |
| Samp. no. | 11A | 12A | 13A | 14A | 15A | 17A | 18A | 19A |
| Alum, ppm | 5·3 | 4·0 | 3·3 | | 4·7 | 4·7 | 2·4 | 13·0 |

*Chart 2.*

| Samp. no. | 19A | 19B | 19C | 19D | 19E | 19F |
|---|---|---|---|---|---|---|
| Alum, ppm | 13·3 | 17·9 | 11·0 | 13·3 | 13·2 | 9·0 |
| Samp. no. | 19G | 19H | 19I | 19J | 19K | |
| Alum, ppm | 14·2 | 6·0 | 10·1 | 10·3 | 8·9 | |

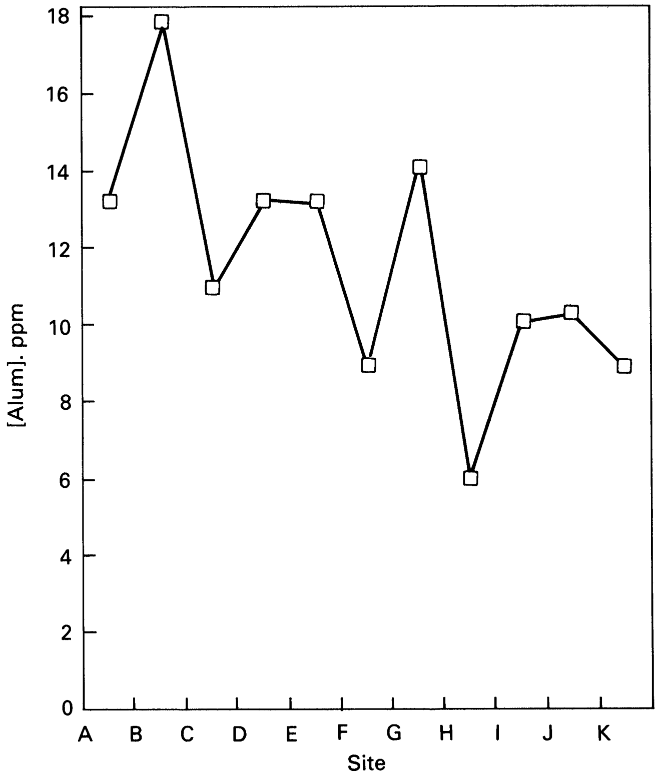

*Figure 2. Variation of alum concentration with the site of collection.*

In existing FIA methodologies for the determination of aluminium, Wyganowski *et al.* [12] used a reagent in 60% ethanol to produce calibrations of 0–0·3 (0–5·31) and 0–0·1 ppm Al (0–1·77 ppm alum). In his comparison of various organic reagents for the flow injection determination of Al, Royset [13] had not provided any linearity data. Royset revealed that the aluminon method was able to tolerate up to 500 ppm P without causing significant interference. Unfortunately, this method was unsuitable for $Al^{3+}$ concentrations of less than 100 ppb. Fluoride can only be tolerated at levels of <0·2 ppm in all the methods. The same author used pyrocatechol violet [14] and obtained linear calibration from 0·1–3 ppm Al (1·77–53·1 ppm alum) upon 70 and 200 microlitre injections. The linear range was extended, presumably from 0·1 ppm of Al, to 10 ppm Al (177 ppm alum) when 10 microlitre was injected. A 6-m coil and a 30 s reaction time was employed. Furthermore, the PCV mixed reagent was unstable for periods in excess of two hours. Bouzid and Macdonald [15] abandoned this system on the basis of its narrow operational pH range, 6·0 ± 0·1 pH unit. They instead opted for the CAS/CPC/ethanol system. The buffer that they had used unusually comprised of hexamine/ammonia, in place of the commonly used hexamine/hydrochloric acid to keep the pH at around 6. A linear range of 0–0·4 ppm Al (0–7·08 ppm alum) was obtained with a limit of detection of 5 ppb Al (88·5 ppb alum). For the calibration from 0 to 0·2 ppm Al (0–3·54 ppm alum), a slope of 28·8 AU/ppm was obtained, yielding a possible signal of >5·7 AU at the top of the calibration range. The sample throughput was ~45 $h^{-1}$.

Henshaw *et al.* [16] again used PCV as the reagent. They obtained a linear calibration of up to 1·0 ppm Al (17·7 ppm alum). Unlike the other methods [12–15], they chose not to acidify the sample and the carrier in order to maintain the sample pH. They have proved that this treatment is quite feasible for the determination of monomeric aluminium species. The strangest result of all appeared in their interference studies. Fluoride would normally cause negative interference for the reason stated above. In their studies, a 0·07 ppm of fluoride spiked into a 0·1 ppm aluminium solution gave a recovery of 110% of aluminium whereas a 0·7 ppm fluoride spiked into a solution of the same concentration caused only 1% positive interference. This appears to suggest that fluoride has an inhibitory effect at lower concentrations but once this has been exceeded, the level of interference is reduced.

### Conclusion

The present automatic flow analyser offers increased performance compared with other FIA instruments in terms of calibration range, precision and sample throughput for the determination of aluminium. The sensitivity of the assay and its ability to cope with interferents is also as good as existing instruments. In these experiments the versatility of the prototype has been demonstrated by its success in analysing concentrations of aluminium up to exceptional levels and its ability to cope with intensely coloured reagents.

### Acknowledgements

The authors would like to thank Biotech Instruments for financial support.

### References

1. Epstein, S. G., *Environmental Geochemistry and Health,* **12** (1990), 65.
2. Driscoll, C. T. and Schecher, D., *Environmental Geochemistry and Health,* **12** (1990), 28.
3. Day, J. P., *Environmental Geochemistry and Health,* **12** (1990), 75.
4. Bertsch, P. M., *Environmental Geochemistry and Health,* **12** (1990), 7.
5. Burger, K., *Organic Reagents in Metal Analysis,* Pergamon, Oxford, 1973.
6. Dougan, W. K. and Wilson, A. L., *Analyst,* **99** (1974), 413.
7. Zhu, Y., Zhang, L. and Li, J., *Talanta,* **30** (1983), 291.
8. Marczenko, Z. and Jarosz, M., *Analyst,* **107** (1982), 1431.
9. Liu, S., *Analyst,* **107** (1982), 428.
10. Sampson, B. and Fleck, A., *Analyst,* **109** (1984), 369.
11. Reis, B. F., Bergamin Filho, H., Zagatto, E. A. G. and Krug, F. J., *Analytica Chimica Acta,* **107** (1979), 309.
12. Wyganowski, C., Motomizu, S. and Toei, K., *Analytica Chimica Acta,* **140** (1982), 313.
13. Royset, O., *Analytica Chimica Acta,* **178** (1985), 223.
14. Royset, O., *Analytica Chimica Acta,* **185** (1986), 75.
15. Bouzid, B. and Macdonald, A. M. G., *Analytica Chimica Acta,* **207** (1988), 337.
16. Henshaw, J. M., Lewis, T. E. and Heithmar, F. M., *International Journal of Environmental Analytical Chemistry,* **34** (1988), 119.
17. Malcolme-Lawes, D. J. and Pasquini, C., *Journal of Automatic Chemistry,* **10** (1988), 192.
18. Tyson, J. F., *Analytica Chimica Acta,* **179** (1986), 131.

